# Stimulus modality influences session-to-session transfer of training effects in auditory and tactile streaming-based P300 brain–computer interfaces

**DOI:** 10.1038/s41598-020-67887-6

**Published:** 2020-07-17

**Authors:** P. Ziebell, J. Stümpfig, M. Eidel, S. C. Kleih, A. Kübler, M. E. Latoschik, S. Halder

**Affiliations:** 1 Institute of Psychology, University of Würzburg, Würzburg, Germany; 2 Institute of Computer Science, University of Würzburg, Würzburg, Germany; 3 School of Computer Science and Electronic Engineering (CSEE), University of Essex, Colchester, UK

**Keywords:** Brain-machine interface, Amyotrophic lateral sclerosis

## Abstract

Despite recent successes, patients suffering from locked-in syndrome (LIS) still struggle to communicate using vision-independent brain–computer interfaces (BCIs). In this study, we compared auditory and tactile BCIs, regarding training effects and cross-stimulus-modality transfer effects, when switching between stimulus modalities. We utilized a streaming-based P300 BCI, which was developed as a low workload approach to prevent potential BCI-inefficiency. We randomly assigned 20 healthy participants to two groups. The participants received three sessions of training either using an auditory BCI or using a tactile BCI. In an additional fourth session, BCI versions were switched to explore possible cross-stimulus-modality transfer effects. Both BCI versions could be operated successfully in the first session by the majority of the participants, with the tactile BCI being experienced as more intuitive. Significant training effects were found mostly in the auditory BCI group and strong evidence for a cross-stimulus-modality transfer occurred for the auditory training group that switched to the tactile version but not vice versa. All participants were able to control at least one BCI version, suggesting that the investigated paradigms are generally feasible and merit further research into their applicability with LIS end-users. Individual preferences regarding stimulus modality should be considered.

## Introduction

P300 brain–computer interfaces (BCIs) provide users with a non-muscular way of communication using control signals recorded via electroencephalography (EEG) and have been successfully applied to various applications such as word spelling, smart home use, brain painting or gaming^1,2^. To this end, they primarily rely on event-related potentials (ERPs), in particular, the eponymous P300 component that is elicited by meaningful, unpredictable and rare stimuli in an oddball paradigm, henceforth referred to as target stimuli, as compared to the frequent irrelevant non-target stimuli^3–5^. The classic way of eliciting these P300 ERPs in BCIs is via visual stimulation, which has been shown to be easily applicable without requiring considerable training efforts to achieve high-speed communication^6–8^.

However, since a main target group for BCIs consists of patients suffering from locked-in syndrome (LIS), a severe form of paralysis that can lead to drastic problems regarding vision, the limits of the common visual P300 BCI have been pointed out and various alternatives of P300 eliciting stimulation approaches have been developed^9–11^. Among those approaches, auditory and tactile P300 BCIs have become a recent focus of research^12–15^. An auditory multi-class BCI using spatially distributed animal sounds has been proven feasible for healthy participants and furthermore, first evidence of successful use by paralyzed patients has been reported^16–18^. Considering tactile approaches, studies involving a multi-class P300 BCI to achieve wheelchair control via vibrotactile stimulations on body parts to steer the wheelchair reported promising results with healthy participants of various age groups as well^19–21^. User performance in these studies could on average be increased by training, yet, cases of BCI-inefficiency were reported even after several sessions of training, and a relatively high workload has been identified as a potential problem in auditory BCI approaches^22–24^. The tactile P300 BCI wheelchair paradigm still needs to be tested with LIS patients and workload-related issues might also be relevant regarding the tactile modality^21^.

The auditory streaming-based paradigm, as another example of a vision-independent BCI, allows only two choices and is therefore a less complex paradigm that aspires to reduce complexity further by a streaming-based stimulus presentation^25,26^. In this streaming-based paradigm, there is not only one sequence of stimuli as in the aforementioned P300 BCI examples, but rather two simultaneous sequences of stimuli, or in other words two stimulus streams, that were arranged to facilitate target selection with each stream presented to one ear only. The stimuli in each stream were made to sound relatively similar to each other by using similar words such as “nope” (target in left ear stimulus stream) and “no” (non-target in left ear stimulus stream) or “yep” (target in right ear stimulus stream) and “yes” (non-target in right ear stimulus stream) and spoken by different voices (male and female). Compared to earlier paradigms, there was no need to remember an abstract meaning of a stimulus, for example a row or a column in a non-visual P300 spelling matrix—if participants wanted to communicate negation, they simply concentrated on the left ear male voice stimulus stream and on the target word “nope”. If participants wanted to communicate affirmation, they simply concentrated on the right ear female voice stimulus stream and the target-word “yep”. So far, first encouraging results with healthy participants and two additional LIS end-users have been reported, suggesting that the streaming paradigm could be a feasible low workload alternative^25,26^.

The major aim of the current study was to replicate and further explore this auditory streaming-based P300 BCI and in addition to that, since the streaming paradigm has not yet been tested with tactile stimuli, to design and test a two-class tactile streaming-based P300 BCI. Since this is our first study involving this streaming approach, we focused on healthy participants and since we were interested in two BCI versions, we also aimed at providing further information on comparisons of auditory and tactile stimuli^27,28^. More specifically, the aims of our study were, first, to determine if a successful use of our auditory/tactile streaming-based P300 BCIs was already possible in the first session of use (defined as online-accuracy above chance level^29^) and second, whether further training sessions lead to a performance increase via training effects (auditory/tactile). Third, to explore whether a cross-stimulus-modality transfer of performance from auditory stimulus modality to tactile stimulus modality or vice versa was possible, which has not been examined before. This is of interest, since a cross-stimulus-modality relation between performances has been shown in visual and auditory P300 BCIs and such a cross-stimulus-modality transfer might also be existent for auditory and tactile BCIs^30^. Fourth, we wondered if it was possible to provide and maintain sufficient motivation throughout our study, since it included several sessions and the streaming-paradigm with its two choices might become monotonous and therefore lead to a decline of motivation. This incident was partially reported by earlier training studies, considering that the importance of motivation has been pointed out by further studies as well^16–21,31,32^.

To ensure heightened and consistent motivation, we took inspiration from recent literature highlighting potential pitfalls and flaws in BCI training studies and decided to integrate gamification aspects based on a “Star Wars” theme in our study^2,33–36^. Outcome measures were selected according to the user-centered design usability criteria to evaluate BCI-controlled applications, namely effectiveness, efficiency and satisfaction^37,38^. Eventually, since earlier findings emphasize the importance of individual preferences in addition to analyzing group means^39,40^ and since we were interested in evaluating potential sources of BCI-inefficiency in our study, we included a closer look on an individual level.

## Methods

### Participants

Healthy participants (*N* = 20; age in years *M* = 25.30, *SD* = 5.17, range 20 to 41; 11 female = 55%) were recruited in Würzburg, Germany (exclusion criteria: auditory or neurological impairments, use of psychotropic substances, left–right disorientation, previous BCI use). All participants spoke German at native-speaker level. Informed consent was obtained from all participants and they were compensated financially. The experimental protocol was conducted in accordance with the ethical guidelines of the Declaration of Helsinki^41^ and approved by the Ethics Committee of the Psychological Institute of the University of Würzburg (approval number: GZEK 2013–11).

### Data collection

A g.USBamp amplifier and 16 Ag/AgCl active g.Ladybird electrodes on a g.Gamma cap (g.tec Medical Engineering GmbH, Schiedlberg, Austria, https://www.gtec.at/) were used for EEG recording: AF7, Fpz, AF8, F3, Fz, F4, FC3, FCz, FC4, C3, Cz, C4, CP3, CPz, CP4, Pz (10–20-system, modified international standard^42^) with the reference electrode attached to the right earlobe and the ground electrode to AFz. The signal was sampled at a rate of 256 Hz with bandpass-filtering (0.1 to 30 Hz, order 8) as well as notch-filtering (48 to 52 Hz, order 4) using a Chebyshev type causal filter via the internal digital filters of the g.USBamp. Data recording, signal processing and stimulus presentation was handled by BCI2000^43^ (running on a Hewlett–Packard ProBook 6460b with Dual-Core-CPU, 4 GB RAM and 64-Bit Windows 7). This EEG data formed the basis for calculating objective BCI usability measures covering effectiveness and efficiency.

To evaluate BCI use on a subjective level, several questionnaires were included. The *Questionnaire for Current Motivation in Learning and Performance Situations* was used in its adapted BCI-version (QCM-BCI) to measure task-related motivation^44,45^*.* The QCM-BCI covers four factors of motivation in learning and performance situations: interest, mastery confidence, incompetence fear and challenge, each with a 7-point Likert-type scale, score range from 1 (“does not apply”) to 7 (“does apply”). Subjective workload as an aspect of efficiency was measured via *NASA-Task Load Index* (NASA-TLX) global workload score, reaching from 0 (lowest subjective workload) to 100 (highest subjective workload)^46,47^. A set of *visual analogue scales* (VAS) was used for operationalizing satisfaction (“satisfaction with BCI system”) and subjective workload (“BCI-control-difficulty”) as well as global motivation. All VAS items were designed as 10 cm lines with one extremum at 0 cm (“not at all”) and the other extremum at 10 cm (“extraordinarily high”). In addition to the VAS, an open question was included to give participants the possibility for concluding remarks on problems and suggestions for BCI improvement.

### Streaming-based P300 BCIs with auditory and tactile stimulation

**Figure 1:**
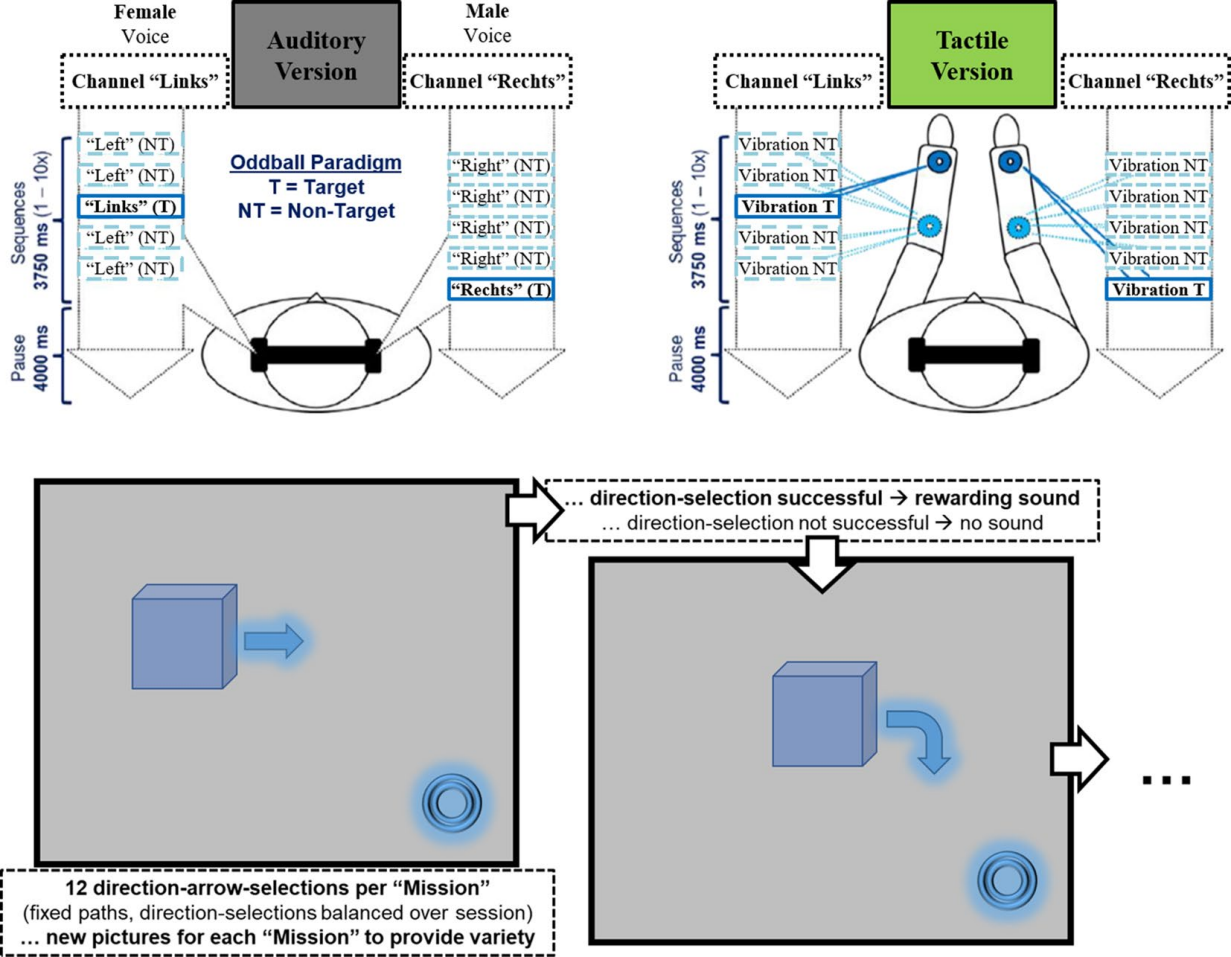
Top: Streaming-based P300 BCIs with auditory stimulation (top left) and tactile stimulation (top right). Bottom: “Star Wars Mission” task where participants had to move objects (symbolized by a cube) towards a goal (ring) along predetermined direction arrows before various backgrounds. The objects and backgrounds that have been used in this study are not shown in this figure due to trademarking issues. Direction arrows were varied but always clearly pointed towards either left or right.

The auditory streaming-based P300 BCI version worked almost exactly as described earlier, the only difference being left-vs.-right-decisions instead of yes-vs.-no-decisions^26^ (Fig. 1). Since we collected data from a German-speaking sample, the German word “rechts” (English meaning “right”) served as a target stimulus on the right ear stimulus stream and the English word “right” served as a non-target stimulus. Similarly, the words “links” and its English translation “left” served as target and non-target stimuli, respectively. Stimuli were presented via stereo-headphones (Sennheiser HD 280 PRO, Sennheiser electronic GmbH & Co. KG, Wedemark, Germany, https://de-de.sennheiser.com/hd-280-pro). Headphone volume was adjusted for each participant, so that all stimuli were pleasantly audible and of similar saliency. Apart from the described differences, all parameters were adopted from Hill and colleagues^26^.

The tactile version was inspired both by the auditory version and by earlier tactile P300 BCIs^19–21,26^ (Fig. 1). The two stimulus streams consisted of the participant’s inner side of the forearms, utilizing tactile vibration stimulators (C-2 Tactors, Engineering Acoustics Inc, Casselberry, FL/USA, https://www.eaiinfo.com/). One tactile stimulator was placed near the wrist of each forearm and defined as the provider of this side’s target stimuli. Another tactile stimulator was placed near the elbow of each forearm and defined as the provider of this side’s non-target stimuli. All four of these tactile stimulators’ vibrations had a duration of 125 ms and an inter-stimulus-interval of 250 ms from the end of one stimulus to the beginning of the next. Stimulus strength was again adjusted for each participant. To ensure comparability, all other parameters were also adopted from the earlier work^26^. Other design options would have been possible for a tactile streaming-based P300 BCI, for example by using different vibration frequencies to discriminate between target and non-target stimuli instead of different locations. However, since earlier work on tactile P300 BCIs highlighted the importance of spatial discriminability of the stimuli^12,13,19,20^, we decided to focus on that aspect in order to avoid introducing any further features in this first version of our tactile streaming-based P300 BCI.

Each stimulus-selection-sequence consisted of ten stimuli in total (one target and four non-target stimuli per stimulus stream). Streams were arranged in constant anti-phase: The stimuli of the second channel started 375 ms after the corresponding stimuli of the first channel, leading to the length of each stimulus-selection-sequence being 3.75 s^48^. The user’s task for both paradigms was to correctly select the movement direction of an object on a computer screen towards a target direction (Fig. 1).

### Training protocol

**Figure 2:**
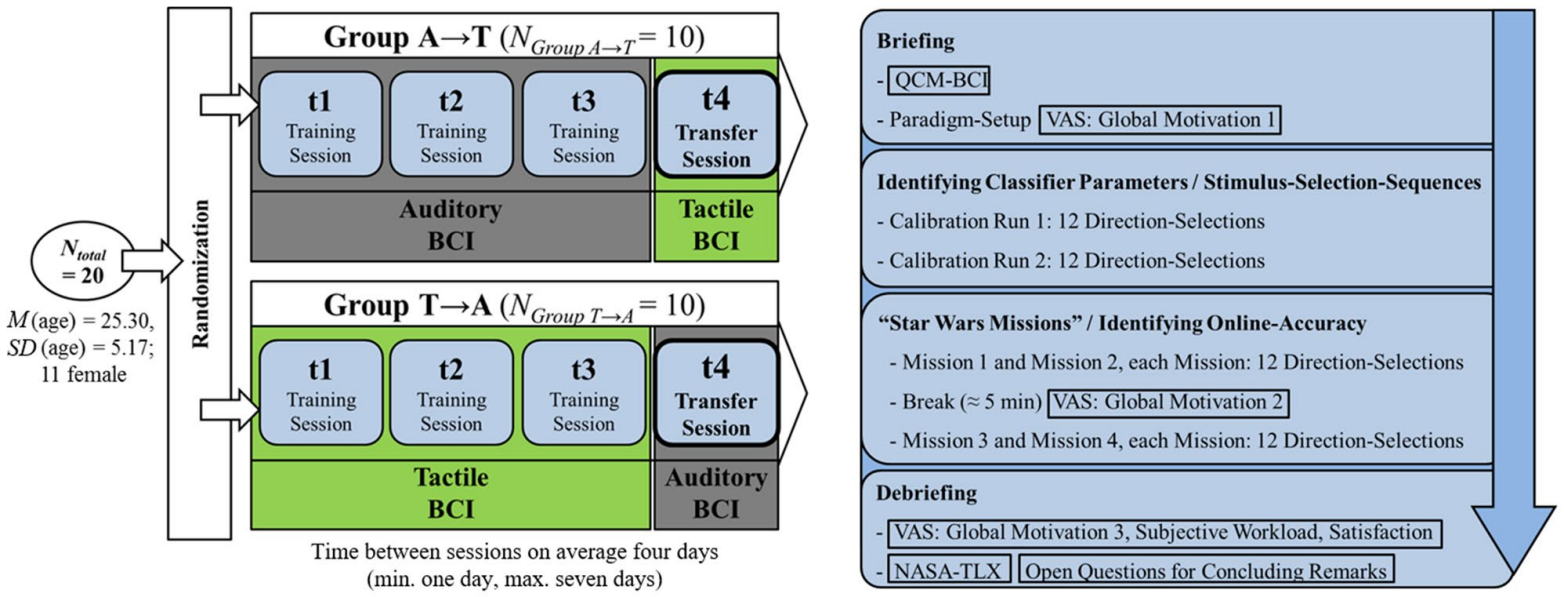
Left: Training overview. Right: Session overview including questionnaires (QCM-BCI, NASA-TLX, VAS).

The training protocol was based on the earlier training studies and suggestions on study design^16,19,33,34^. The user’s task was embedded in three training sessions (*t1*, *t2* and *t3*) and one subsequent transfer session (*t4*). Participants were randomized into two groups of 10. Group A used the auditory BCI version in the training sessions and transferred to the tactile version at t4. Group T used the tactile version in the training sessions and transferred to the auditory version at t4. Time between sessions was four days on average (min. one day, max. seven days). Each session consisted of the following four parts (Fig. 2).

First, users were given psychological questionnaires (QCM-BCI; VAS: global motivation 1) and briefed carefully on relevant information for BCI (e.g. avoidance of artifacts caused by unnecessary muscular activity). Two strategies were recommended: focusing on the relevant stimulus stream (and ignoring the irrelevant), as well as counting the target-stimuli of the relevant stimulus stream to focus attention. Then, the paradigm was set up; EEG, headphones, comfortable position in front of the computer screen and tactile stimulators if the tactile version was used. Second, the classifier was individually calibrated to estimate the optimal classification weights for EEG signal classification. To this end, 24 direction-selections were performed (split in two calibration runs of 12 direction-selections), each involving 10 stimulus-selection-sequences. The classification weights and therefore the number of stimulus-selection-sequences for the upcoming online-classification runs were set such that 70% offline-accuracy was reached (based on the calibration data) plus three additional stimulus-selection-sequences. For instance, if 70% offline-accuracy was reached with three stimulus-selection-sequences, six stimulus-selection-sequences were configured for the online-classification runs. This heuristic was identical to earlier studies^18^. Third, four online-classification runs including 48 direction-selections followed (12 direction-selections per run), again including a short break with a questionnaire after two runs (VAS: global motivation 2). Fourth, the concluding set of psychological questionnaires followed (VAS: global motivation 3, satisfaction, subjective workload; NASA-TLX; open questions for concluding remarks) and participants were carefully debriefed, including the opportunity to ask further questions or to discuss potential problems.

Direction-selections were counterbalanced over all calibration runs as well as online-classification runs. To give immediate feedback on successful direction-selection, a one-second long feedback sound was given via the headphones after each correct choice. No feedback sound was given for an incorrect choice. As elaborated before, a “Star Wars” theme was incorporated in the task, a rewarding beeping sound of a “Star Wars” robot was chosen for the feedback after a correct selection and various pictures were used to recreate scenes from “Star Wars”.

### Data analysis

IBM SPSS Statistics 23 (IBM, Armonk, NY/USA, https://www.ibm.com/analytics/de/de/technology/spss/) was used for statistical calculations. Analyses of variance (ANOVAs) were conducted to examine the training effects (t1, t2 and t3) and followed up by post-hoc t-tests and t-tests to examine cross-stimulus-modality transfer effects (t1 vs. t4 and t3 vs. t4) for each group of the dependent variables: EEG, objective and subjective BCI usability as well as motivation (alpha-error-level conventionally p = 0.05; Bonferroni–Holm correction against multiple comparisons alpha-inflation; marginally significant findings and descriptive tendencies explored to take relatively small sample size into account). Cohen’s *d* is reported as an effect size measure for t-tests and *η*^*2*^_*p*_ for ANOVAs. To facilitate interpretation of the effect size Cohen’s *d*, it was calculated so that positive values indicate a change in the direction that was intended by the experimental design, e.g. an increase in performance or a decrease in subjective workload (and negative values a change in the direction that was not intended, e.g. a decrease in motivation)^29,49–52^.

EEG data were analyzed with the MATLAB-Toolbox EEGLAB and MATLAB scripts^53^. Data epoch segments ranged from 0 to 800 ms after stimulus-presentation, whereas − 100 ms to 0 ms before stimulus presentation served for baseline correction. Epochs related to target stimuli were grouped and averaged; the same procedure was applied to the non-target stimuli. A stepwise linear discriminant analysis (SWLDA) was applied to the data from the calibration runs to determine the weights and consequently the number of necessary stimulus-selection-sequences for online run signal classification according to the heuristic described in the section “training protocol” (number of stimulus-selection-sequences needed to achieve 70% offline-accuracy based on the calibration data plus three additional stimulus-selection-sequences). A description of SWLDA is not given here due to spatial limitations, but can be found in earlier work that focuses on SWLDA more specifically^54^. ANOVAs were calculated for both the *P300 amplitude*, defined as highest positive peak between 200 and 700 ms post-stimulus, as well as the *P300 latency*, the time interval between stimulus presentation and P300 amplitude. Since highest amplitudes were measured at Cz, analysis focused on Cz.

*Online-accuracy* and *information transfer rate* (*ITR*) were calculated as objective measures and analyzed with ANOVAs. Online-accuracy (effectiveness—how accurate users can accomplish a task^37^) was defined as the percent value of correct selections out of all selections of a session. As a measure of BCI-inefficiency, we furthermore analyzed how many participants could significantly exceed the chance threshold of 63.59%^29^. ITR (efficiency—invested costs of the user in relation to achieved effectiveness^37^) was defined as the amount of correctly transferred information during the time interval of one minute, using the following formula (1)^55^:

1 $${\it {B }} = {\it \log}_{{2}} {\it {N }} + {\it { P }}*{\it \log}_{{2}} {\it {P }} + \, \left( {{1 }{-}{\it { P}}} \right) \, *{\log}_{{2}} \frac{1 - P}{{N - 1}}$$

With *B* standing for bits per selection, *N* standing for number of possible selection-targets and *P* standing for the estimated probability of a correct classification, based on the empirically found online-accuracy. To calculate the ITR, B was multiplied with the number of possible selections per minute (*SPM*), using the following formula (2), with *S* standing for the number of stimulus-selection-sequences that was chosen for each participant at each session and taking into account the duration of each stimulus-selection-sequence (3.75 s) as well as the post-stimulus-selection-sequence break (4 s):

2 $${\it {SPM }} = \frac{60s}{{S * 3.75s + 4s}}$$

To give insight on a subjective level into efficiency, satisfaction and motivation, the quantitative questionnaires were evaluated with ANOVAs (QCM-BCI: challenge, incompetence fear, interest, mastery; NASA-TLX: subjective workload; VAS: global motivation (mean value of 1, 2 and 3 for each session), subjective workload, satisfaction). Finally, the answers to the concluding open questions were sighted.

## Results

A summary of the significant within-group post-hoc t-tests to examine training effects can be found in Table 1, a summary of the significant within-group t-tests to examine cross-stimulus-modality transfer effects in Table 2.

**Table 1:** Summary of significant within-group post-hoc t-tests to examine training effects, after significant effects were found in the 2 (group: A, T) × 3 (training session: t1, t2, t3) ANOVAs.

**Comparison: pre vs. post**	**Group (*N*)**	***M***_***pre***_	***SD***_***pre***_	***M***_***post***_	***SD***_***post***_	***t (df)***	***p***	***d***
Electroencephalography: P300 Amplitude (µV)								
t1 vs. t2	A (10)	5.78	2.18	7.87	3.42	− 3.74 (9)	0.005	0.96
t1 vs. t3	A (10)	5.78	2.18	7.48	4.48	− 1.95 (9)	0.084^†^	0.78
Objective BCI Usability Efficiency: ITR (bits/min)								
t1 vs. t2	A + T (20)*	0.95	0.72	1.36	0.81	− 2.78 (19)	0.012	0.57
t1 vs. t3	A + T (20)*	0.95	0.72	1.63	1.07	− 3.23 (19)	0.004	0.94
Subjective BCI Usability Efficiency: NASA-TLX: Global Workload (0: “lowest”–100: “highest”)								
t1 vs. t3	A (10)	54.97	5.98	41.80	6.66	3.10 (9)	0.013	2.20
t2 vs. t3	A (10)	50.30	5.71	41.80	6.66	2.88 (9)	0.018	1.49
Subjective Motivation: VAS: Global Motivation (0: “not at all”–100: “extraordinarily high”)								
t1 vs. t3	T (10)	85.50	8.49	77.77	12.87	4.58 (9)	0.001	− 0.91

No significant ANOVA effects and not further tested or no significant/no marginally significant post-hoc test differences for: P300 Latency (Electroencephalography)—Online-Accuracy (Objective BCI Usability Effectiveness)—VAS: Subjective Workload (Subjective BCI Usability Efficiency)—VAS: Satisfaction (Subjective BCI Usability Satisfaction)—QCM-BCI: Interest, Mastery Confidence, Incompetence Fear, Challenge (Subjective Motivation).

*ANOVA results indicated a significant main effect for the within-factor “training session” (t1, t2, t3) but no group differences, therefore post-hoc t-tests were analyzed for both groups combined in this case.

^†^Post-hoc t-tests that failed to reach significance after Bonferroni–Holm correction as well as post-hoc t-tests that only reached marginal significance (*p* < 0.10) are mentioned, but marked with “†”.

**Table 2:** Summary of significant within-group t-tests to examine cross-stimulus-modality transfer effects.

**Comparison: pre vs. post**	**Group (*N*)**	***M***_***pre***_	***SD***_***pre***_	***M***_***post***_	***SD***_***post***_	***t (df)***	***p***	***d***
Electroencephalography: P300 Amplitude (µV)								
t1 vs. t4	A (10)	5.78	2.18	10.35	4.87	− 3.32 (9)	0.009	2.10
Electroencephalography: P300 Latency (ms)								
t1 vs. t4	T (10)	439.00	115.36	528.40	86.41	− 3.26 (9)	0.010	− 0.77
t3 vs. t4	T (10)	442.50	85.05	528.40	86.41	− 3.91 (9)	0.004	− 1.01
Subjective BCI Usability Efficiency: VAS: Subjective Workload (0: “not at all” – 100: “extraordinarily high”)								
t1 vs. t4	T (10)	30.95	14.58	55.67	19.47	3.87 (9)	0.004	− 1.70
t3 vs. t4	T (10)	33.00	13.29	55.67	19.47	3.02 (9)	0.014	− 1.71
Subjective Motivation: VAS: Global Motivation (0: “not at all”–100: “extraordinarily high”)								
t3 vs. t4	A (10)	79.55	15.23	76.96	17.32	2.43 (9)	0.038	− 0.17
t1 vs. t4	T (10)	85.50	8.49	79.75	9.99	2.32 (9)	0.046^†^	− 0.68
Subjective Motivation: QCM-BCI: Interest (1: “does not apply”–7: “does apply”)								
t3 vs. t4	A (10)	4.48	1.07	4.92	0.77	− 2.58 (9)	0.030	0.41
t3 vs. t4	T (10)	5.48	0.67	5.62	0.65	− 2.09 (9)	0.066^†^	0.21

No significant/no marginally significant differences for: Online-Accuracy (Objective BCI Usability Effectiveness)—ITR (Objective BCI Usability Efficiency)—NASA-TLX: Global Workload (Subjective BCI Usability Efficiency)—VAS: Satisfaction (Subjective BCI Usability Satisfaction)—QCM-BCI: Mastery Confidence, Incompetence Fear, Challenge (Subjective Motivation).

^†^Tests that failed to reach significance after Bonferroni–Holm correction as well as tests that only reached marginal significance (*p* < 0.10) are mentioned, but marked with “†”.

### Electroencephalography

**Figure 3:**
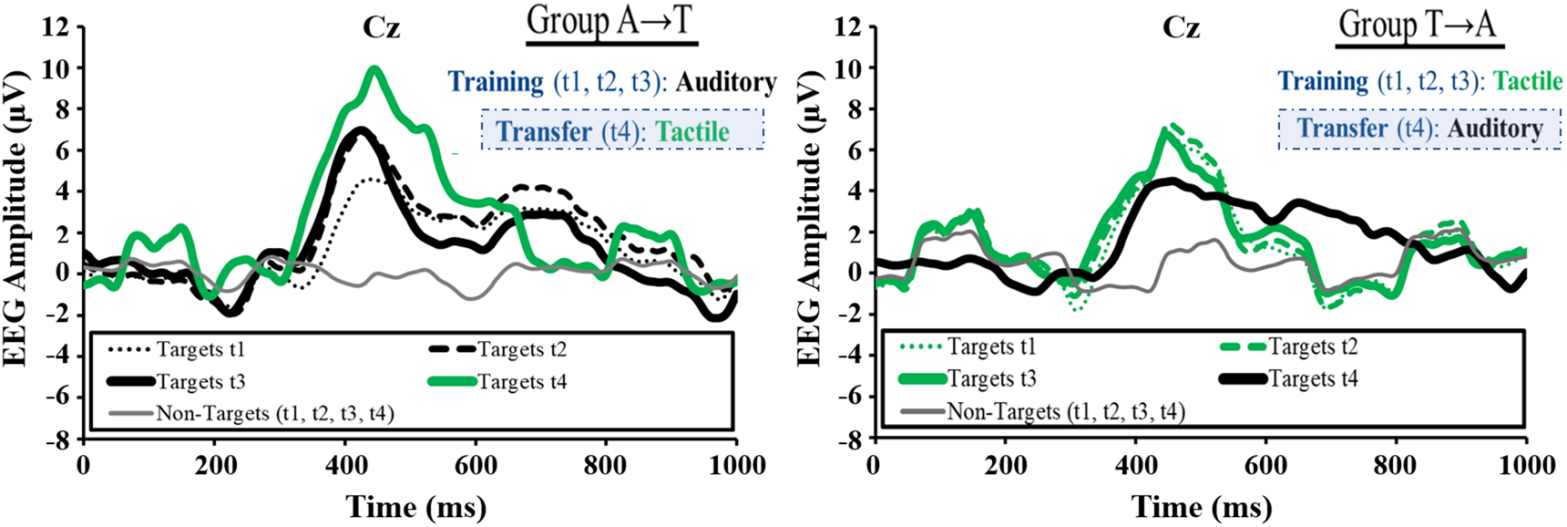
Average electroencephalography (EEG) patterns at Cz for all participants in dependence of stimulus modality and relevance.

Visual analysis of the average EEG patterns at Cz for all participants (Fig. 3) for each session suggests a P300 elicitation in all training sessions (t1, t2, t3) for both the auditory BCI version (group A) and the tactile BCI version (group T) as well as in the cross-stimulus-modality transfer session (t4). Group A showed a descriptive amplitude increase from t1 to t2 that remained stable towards t3 and showed a further increase when switching to the tactile BCI at t4. In contrast, group T remained stable over t1, t2 and t3 and showed a decrease in the cross-stimulus-modality transfer session (switch to the auditory BCI).

The 2 (group) × 3 (training session) ANOVA on P300 amplitudes revealed a significant interaction (*F*(2, 36) = 3.34, *p* = 0.047, *η*^*2*^_*p*_ = 0.156) caused by a significant difference between t1 (*M* = 5.78, *SD* = 2.18) and t2 (*M* = 7.87, *SD* = 3.42) within group A (*t*(9) = − 3.74, *p* = 0.005, *d* = 0.96). Apart from a marginally significant difference between t1 and t3 (*M* = 7.48, *SD* = 4.48) within group A (*t*(9) = − 1.95, *p* = 0.084, *d* = 0.78), post-hoc tests revealed no significant within- or between-group differences. No significant training effects were found in the 2 (group) × 3 (training session) ANOVA on P300 latency.

The t-tests on cross-stimulus-modality transfer effects showed a significant difference in P300 amplitude between t1 and t4 (*M* = 10.35, *SD* = 4.87) within group A (*t*(9) = − 3.32, *p* = 0.009, *d* = 2.10) and a significant P300 latency increase between t1 (*M* = 439.00, *SD* = 115.36) and t4 (*M* = 528.40, *SD* = 86.41) within group T (*t*(9) = − 3.26, *p* = 0.010, *d* = − 0.77) as well as t3 (*M* = 442.50, *SD* = 85.05) and t4 within group T (*t*(9) = − 3.91, *p* = 0.004, *d* = − 1.01). No further significant differences were found.

### Objective measures for BCI usability (effectiveness and efficiency)

**Figure 4:**
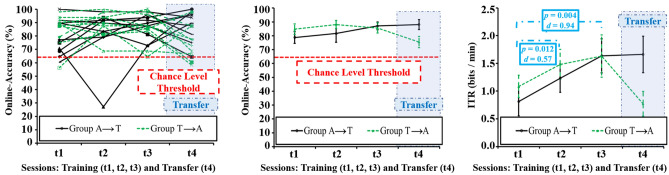
Objective measures for effectiveness (Online-Accuracy) and efficiency (Information Transfer Rate, ITR). Graphs on the left show individual online-accuracies, graphs in the center and on the right show average profiles for online-accuracy and ITR, with error bars marking standard errors of the means and significant p-values (after Bonferroni–Holm correction). Red dotted horizontal line at online-accuracy level of 63.59% indicates the chance level for a two-class BCI with 48 trials and a significance level of α = 0.05^29^.

The 2 (group) × 3 (training session) ANOVA for average online-accuracies as a measure of effectiveness revealed no significant effects, while the 2 (group) × 3 (training session) ANOVA for average ITRs as a measure of efficiency revealed a significant main effect of the training session (*F*(2, 36) = 7.36, *p* = 0.002, *η*^*2*^_*p*_ = 0.290), mean profiles are depicted in Fig. 4. This main effect was caused by a significant increase between t1 (*M* = 0.95, *SD* = 0.72) and t2 (*M* = 1.36, *SD* = 0.81) over both groups (*t*(19) = − 2.78, *p* = 0.012, *d* = 0.57) as well as between t1 and t3 (*M* = 1.63, *SD* = 1.07) over both groups (*t*(19) = − 3.23, *p* = 0.004, *d* = 0.94).

Cross-stimulus-modality transfer effect t-tests on online-accuracy and ITR did not show any significant differences between t1 and t4 or t3 and t4. However, in both online-accuracy and ITR, a descriptive performance decrease could be observed when group T switched to the auditory BCI at t4, while group A performance remained stable. A closer look at individual performances (Fig. 4) further revealed a decrease of online-accuracies in 8 out of 10 participants of group T from t3 to t4, with two participants scoring below the chance level of 63.59%^29^. In comparison, only 5 out of 10 participants of group A showed an online-accuracy decrease from t3 to t4; two of these cases showing only a minor decrease of < 5.00%, with the highest decrease being 16.67%. In contrast, all but one of the decreases of group T were higher than 5.00% between 9.67% up to 31.25%. All participants achieved an online-accuracy above chance level in at least one session, with some participants indicating a preference for one BCI version over the other.

### Subjective measures for BCI usability (efficiency and satisfaction) and motivation

**Figure 5:**
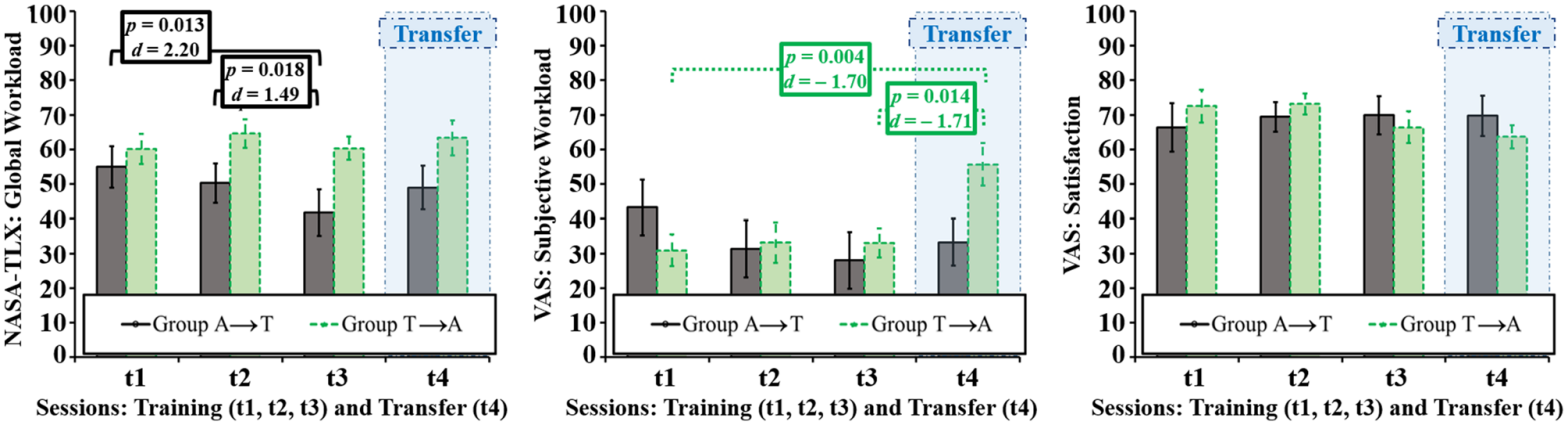
Subjective measures for efficiency (NASA-Task Load Index, NASA-TLX: Global Workload; Visual Analogue Scales, VAS: Subjective Workload) and satisfaction (VAS: Satisfaction). Graphs show average profiles with error bars marking standard errors of the means and significant p-values (after Bonferroni–Holm correction).

Subjective workload as measured with the NASA-TLX global workload score and a VAS on BCI-control-difficulty, decreased in the training sessions in group A but remained stable in group T (Fig. 5). This decrease did not manifest as a significant effect in the 2 (group) × 3 (training session) ANOVA including the VAS, but in a significant interaction in the 2 (group) × 3 (session) ANOVA including the NASA-TLX global workload score (*F*(2, 36) = 3.43, *p* = 0.043, *η*^*2*^_*p*_ = 0.160). This interaction was caused by significant differences between t1 (*M* = 54.97, *SD* = 5.98) and t3 (*M* = 41.80, *SD* = 6.66) within group A (*t*(9) = 3.10, *p* = 0.013, *d* = 2.20) and t2 (*M* = 50.30, *SD* = 5.71) and t3 within group A (*t*(9) = 2.88, *p* = 0.018, *d* = 1.49). No significant change occurred in the VAS on user satisfaction, however, a satisfaction increase by trend is visible in group A, contrary to a decrease by trend in group T (Fig. 5). In terms of cross-stimulus-modality transfer effects, significant differences occurred for the VAS on subjective workload between t1 (*M* = 30.95, *SD* = 14.58) and t4 (*M* = 55.67, *SD* = 19.47) within group T (*t*(9) = − 3.87, *p* = 0.004, *d* = − 1.70) and t3 (*M* = 33.00, *SD* = 13.29) and t4 within group T (*t*(9) = − 3.02, *p* = 0.014, *d* = − 1.71). Apart from these results, no significant differences between t1 and t4 or t3 and t4 were found for the efficiency and satisfaction measures.

**Figure 6:**
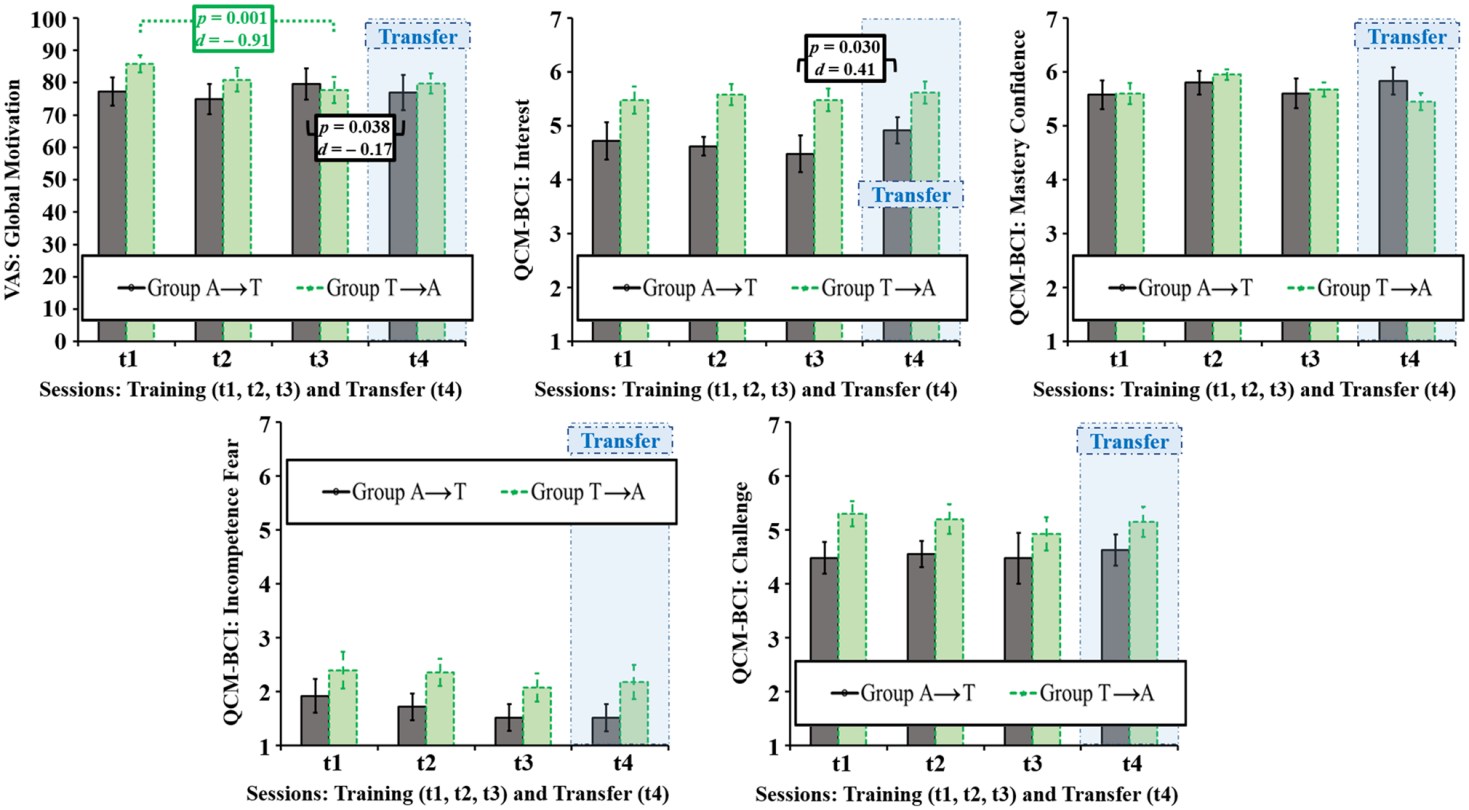
Subjective measures for motivation (Visual Analogue Scales, VAS: Global Motivation; Questionnaire for Current Motivation in Learning and Performance Situations BCI-version, QCM-BCI: Interest, Mastery Confidence, Incompetence Fear, Challenge). Graphs show average profiles with error bars marking standard errors of the means and significant p-values (after Bonferroni–Holm correction).

The 2 (group) × 3 (training session) ANOVA for the VAS on global motivation revealed a significant interaction effect (*F*(2, 36) = 6.13, *p* = 0.005, *η*^*2*^_*p*_ = 0.254), caused by a decrease in global motivation in group T (Fig. 6). Even though average global motivation was on a relatively high level between 74.93% and 85.80% during all training sessions among both groups, only group A showed no significant differences between training sessions, while a significant difference manifested between t1 (*M* = 85.50, *SD* = 8.49) and t3 (*M* = 77.77, *SD* = 12.87) within group T (*t*(9) = 4.58, *p* = 0.001, *d* = − 0.91). Furthermore, a significant cross-stimulus-modality transfer difference was found between t3 (*M* = 79.55, *SD* = 15.23) and t4 (*M* = 76.96, *SD* = 17.32) within group A (*t*(9) = 2.43, *p* = 0.038, *d* = − 0.17), indicating a decrease in global motivation when switching from the auditory to the tactile BCI. No significant cross-stimulus-modality transfer difference was measured between t1 and t4 (*M* = 79.75, *SD* = 9.99) or t3 and t4 for group T with the difference between t1 and t4 not reaching significance after Bonferroni–Holm correction (*t*(9) = 2.32, *p* = 0.046, *d* = − 0.68). A deeper exploration of motivation with 2 (group) × 3 (training session) ANOVAs on task-related motivation, as measured by the four aspects covered by the QCM-BCI (interest, mastery confidence, incompetence fear and challenge), revealed no significant changes over training sessions. Even though interest and mastery confidence remained stable and incompetence fear decreased by trend for both groups A and T, a group difference by trend was visible regarding challenge (Fig. 6). While group A remained stable between t1 (*M* = 4.48, *SD* = 0.93), t2 (*M* = 4.55, *SD* = 0.77) and t3 (*M* = 4.48, *SD* = 1.49), group T showed a decrease by trend from t1 (*M* = 5.30, *SD* = 0.74) and t2 (*M* = 5.20, *SD* = 0.87) towards t3 (*M* = 4.93, *SD* = 0.98). A significant difference in interest occurred between t3 (*M* = 4.48, *SD* = 1.07) and t4 (*M* = 4.92, *SD* = 0.77) within group A (*t*(9) = − 2.58, *p* = 0.030, *d* = 0.41), the comparison between t3 (*M* = 5.48, *SD* = 0.67) and t4 (*M* = 5.62, *SD* = 0.65) was marginally significant within group T (*t*(9) = − 2.09, *p* = 0.066, *d* = 0.21). Other than these, no significant differences in comparison to the cross-stimulus-modality transfer session manifested between t1 and t4 or t3 and t4 in the QCM-BCI aspects of task-related motivation.

The answers to the open questions on concluding remarks revealed individual differences, with some participants reporting they were comfortably able to operate both BCI versions and others reporting a preference towards either the auditory or the tactile BCI version.

## Discussion

A successful use of the streaming-based P300 BCI with an online-accuracy above chance level (of 63.59%^29^) was possible in the first session for 9 out of 10 auditory BCI users as well as 9 out of 10 tactile BCI users. The average online-accuracy lay significantly above chance level for both groups (auditory: 78.75%, tactile: 84.79%). Online-accuracy did not significantly increase via training, but ITR as an objective performance measure of efficiency increased significantly for both groups. A significant increase of P300 amplitude and a significant decrease in subjective workload, as a measure for subjective efficiency, was visible for the auditory BCI only. The auditory BCI group further reported an increase by trend regarding satisfaction, which remained absent in the tactile BCI group, where satisfaction even declined by trend. Overall, this indicates the value of training for both groups, but shows stronger evidence for the auditory BCI group and comparably more stagnation in the tactile BCI group. Regarding cross-stimulus-modality transfer of performance in the cross-stimulus-modality transfer session, evidence was found for switching from the auditory to the tactile BCI but not vice versa. To emphasize, when the auditory group switched to the tactile BCI, P300 amplitude significantly increased, whereas P300 latency, online-accuracy, ITR, subjective workload as well as satisfaction remained stable. Conversely, when the tactile group switched to the auditory BCI, P300 latency and subjective workload increased significantly; P300 amplitude, online-accuracy as well as ITR decreased by trend, while satisfaction remained stable. Every participant was able to use at least one BCI version above chance level and, while on average the cross-stimulus-modality transfer was easier from auditory to tactile, some participants preferred the auditory over tactile BCI and several participants could use both BCI versions equally well. Global motivation remained stable during training in the auditory group, while it declined significantly in the tactile group, with global motivation in both groups still being on a relatively high level (between 85.55% and 74.93%). The feeling of challenge declined by trend in the tactile group only, apart from that, both groups equally showed stable mastery confidence and interest during training as well as a decrease of incompetence fear by trend. When switching BCI versions, mastery confidence, challenge and incompetence fear remained stable in the group switching to the tactile BCI. Interest significantly increased before the actual use during which global motivation significantly declined compared to the training sessions. For the group switching to the auditory BCI, all motivational measures remained stable compared to the first as well as to the last training session.

The ease-of-use in the first session confirms earlier streaming-based P300 BCI findings^25,26^ and the training effects, found in both streaming-based BCI versions, replicate earlier findings with sequential P300 BCI paradigms with auditory as well as with tactile stimuli^16–19^. The possibility of cross-stimulus-modality transfer furthermore adds evidence to earlier work with visual and auditory P300 BCIs^30^, however, our data indicate that the switch from tactile to auditory was more difficult. This difficulty in switching might be explained by the collected psychological measures: The auditory group reported a stable global motivation and a remaining feeling of challenge as well as an experience of decreasing subjective workload going along with their performance training effects. In contrast to that, the tactile group reported a decline in global motivation as well as a slight decline in challenge and no change in subjective workload despite their training effects evident in performance. This might implicate a monotony that was absent in the auditory group, which felt constantly challenged and motivated during training, therefore experiencing more training effects. This monotony might stem from the tactile paradigm being both more intuitive, as it was by trend easier to use in the first session, as well as consequently less complex compared to the auditory paradigm. The auditory paradigm included different voices speaking different words, while in the tactile paradigm all stimuli were exactly the same and only distinguishable by their location on the participants forearms. Regardless, motivation was in general on a relatively high level over the whole study, indicating the usefulness of the aforementioned study design guidelines and the accompanying inclusion of gamification ideas^2,33–36^. This could have helped to prevent participant dropout and signs of motivational decline, which have been reported in earlier studies^16–22^. It is noteworthy that while on some occasions, participants in our study did not perform above chance level, across all sessions every participant managed to control at least one of the BCIs successfully above chance level. For example, one participant managed to score an accuracy of 97.92% in the cross-stimulus-modality transfer session after performing between only 27.08% and 72.92% in the training sessions. Individual preferences like this have been reported in patients before^39,40^ and these encouraging results regarding BCI-inefficiency once more replicate earlier streaming-paradigm findings^25,26^. The difference between average online-accuracy and average ITR underlines the usefulness of exhaustive data collection according to the user-centered design approach: While neither training effects nor cross-stimulus-modality transfer effects are revealed by online-accuracy analysis (measuring the user-centered design criterion effectiveness—how accurate users can accomplish a task^37^), both can be detected via ITR (measuring the user-centered design criterion efficiency—invested costs of the user in relation to achieved effectiveness^37^). Although online-accuracy does not increase significantly, which may be caused by a ceiling effect (which occurred despite our adaptation of stimulus-selection-sequences based on the offline-accuracy reached in the calibration runs, with the intention to allow training effects and to prevent ceiling effects), ITR detects a significant increase because it puts the stable online-accuracy in relation to the increase in possible SPM (which takes into account the required time, depending on the number of stimulus-selection-sequences selected based on the offline-accuracy achieved in the calibration runs, see formula (2)). There seems to be an inconsistency regarding the P300 amplitude results that do not indicate a training effect in the tactile group, while the ITR results do. However, the P300 pattern elicited by the targets basically just needs to be pronounced enough to be clearly discriminative from the EEG pattern elicited by the non-targets. User performance does not necessarily depend on the P300 amplitude of a singular electrode, rather the SWLDA selects the best discriminatory features for each participant based on all electrodes. However, SWLDA signal classification results are not suitable for visualization, which is why we reported the P300 amplitude at electrode Cz, where it was most pronounced on average.

A limiting aspect of the current study is the sample, which has a sample size that is comparable to most of the cited BCI studies but still relatively small and allows only limited conclusions. Especially problematic is the reliability of between-group testing, which led to focusing on within-group comparisons and highlighting individual differences. It is interesting that we find significant effects in such a relatively small sample size; however, a bigger sample size and future studies are needed to validate and expand our findings. The sample also only consisted of healthy participants between 20 and 41 years. To allow better conclusions about LIS end-users, a different age group should be sampled for future studies^19,21^ or actual LIS end-users should be recruited. The current paradigm would then need to be adjusted for LIS end-users who might have vision problems and therefore would experience difficulties in recognizing which direction selection they are supposed to choose, since this information is presented only visually in the current study. Further adjustment would help to optimize SPM, which was limited due to relative conservative parameter settings of the current study (e.g. long pauses and breaks, relatively many non-targets). This in turn led to relatively low ITRs in comparison to earlier work^16–21^. An increase in ITR could, for example, be achieved by reducing the relatively long post-stimulus-selection-sequence break (4 s) or the amount of non-target stimuli (currently eight in each stimulus-selection-sequence) and by adjusting these parameters to the individual user’s needs. Furthermore, the signal processing pipeline could be updated (e.g. implementing shrinkage LDA). Eventually, it has to be emphasized that other versions of a streaming-based tactile P300 BCI might lead to different results than the one used in this study. This could be altered, for example, by using different vibration frequencies of the tactile stimulators to discriminate between targets and non-targets instead of different locations as implemented in this study based on earlier work^12,13,19,20^.

Future studies with both the aforementioned and other recently examined paradigms are planned to be conducted directly with LIS end-users in a training study approach similar to earlier work with sequential auditory paradigms^18^ and eventually in independent home use^56–58^. For this use, the paradigm of the current study just needs to be slightly altered and, instead of left–right-decisions, return to the original idea of yes–no-decisions, where first promising results with two LIS patients have been reported^26^. In addition, the streaming-based P300 BCI might be feasible in further applications, such as in hybrid BCIs as a “switch” or “selector” or by inclusion of an error-correction mechanism based on error-related ERPs^38,59^. Potential monotony, as has occurred in the current study’s tactile paradigm, might be prevented by varying the type of binary decisions (as part of a decision tree with multiple binary decision branches as suggested before^25^). As mentioned previously, it might also be expedient to alter the tactile streaming-based P300 BCI by introducing different frequencies to discriminate between target and non-target stimuli. This could either be an alternative to spatial stimulus discrimination or used in combination with already spatially discriminable stimuli to enhance performance (e.g. the tactile streaming-based P300 used in this study might be improved by adding different vibration frequencies to the already spatially separated targets and non-targets). Adding an additional stimulus stream would also be an option that might be fruitful for studies to come, since streaming-based paradigms with more than two streams have shown encouraging results^60^. Last, but not least, our study points out that different users prefer different stimulus modalities and that the success of cross-stimulus-modality transfer is user-specific and might even be modality-specific in general—yet, it remains unclear why this is the case. Future research is needed to shed more light on this by creating study designs that allow a clearer analysis of why there is no general cross-stimulus-common “BCI skill” by a more detailed examination of factors that contribute to successful BCI use. For example, possible differences in the ability of focusing attention on different target stimuli could be relevant (leading to a more solid P300 component elicitation which could improve BCI performance).

In conclusion, our findings provide further evidence that the streaming-based P300 BCI is a promising vision-independent approach with auditory stimuli as well as with tactile stimuli. The modality differences that we found on the average group level regarding training and cross-stimulus-modality transfer effects as well as individual preferences should be examined in more detail and considered for future applications. Further consideration should be given to the literature on BCI study design, which ensures the possibility of an exhaustive analysis regarding the user-centered design criteria effectiveness, efficiency and satisfaction and might have helped to achieve high motivation of participants in our study and zero dropout.

## Data Availability

The datasets generated during and/or analyzed during the current study are available from the corresponding author on reasonable request.
